# “Won’t get fooled again”: statistical fault detection in COVID-19 Latin American data

**DOI:** 10.1186/s12992-022-00899-1

**Published:** 2022-12-16

**Authors:** Dalson Figueiredo Filho, Lucas Silva, Hugo Medeiros

**Affiliations:** 1 Department of Political Science, Universidade Federal de Pernambuco, Recife, Pernambuco Brazil; 2 Department of Medicine, Universidade Estadual de Ciências da Saúde do Estado de Alagoas, Rua Dr. Jorge de Lima, 113 - Trapiche da Barra, Maceió, Alagoas 57010-300 Brazil

**Keywords:** Public health surveillance, Data reliability, Newcomb-Benford law, Kullback-Leibler divergence, Latin America

## Abstract

**Background:**

Claims of inconsistency in epidemiological data have emerged for both developed and developing countries during the COVID-19 pandemic.

**Methods:**

In this paper, we apply first-digit Newcomb-Benford Law (NBL) and Kullback-Leibler Divergence (KLD) to evaluate COVID-19 records reliability in all 20 Latin American countries. We replicate country-level aggregate information from Our World in Data.

**Results:**

We find that official reports do not follow NBL’s theoretical expectations (*n* = 978; chi-square = 78.95; KS = 4.33, MD = 2.18; mantissa = .54; MAD = .02; DF = 12.75). KLD estimates indicate high divergence among countries, including some outliers.

**Conclusions:**

This paper provides evidence that recorded COVID-19 cases in Latin America do not conform overall to NBL, which is a useful tool for detecting data manipulation. Our study suggests that further investigations should be made into surveillance systems that exhibit higher deviation from the theoretical distribution and divergence from other similar countries.

## Introduction

The SARS-CoV-2 virus has infected almost 630 million people worldwide, and caused approximately 6,5 million deaths as of November 2022 [1]. Unlike previous outbreaks, a distinguishing feature of the COVID-19 epidemic is the unprecedented availability of data [2–4]. However, since the beginning of the SARS-CoV-2 pandemic, much concern has been raised about the epidemiological estimates reliability [5, 6].

Several political leaders challenged the accuracy of COVID-19 reports. In the U.S., the current leading country in total death toll (more than 1 million fatalities as of November 4, 2022), former President Donald Trump repeatedly accused China of data manipulation [7]. In Brazil, the 2nd leading nation in absolute number of deaths (close to 690,000 as of November 4, 2022), President Jair Bolsonaro accused state governors of falsifying data to trick the population and extract public resources [8].

Following Silva and Figueiredo Filho [9], Balashov, Yan and Zhu [10], Koch and Okamura [7], and Kilani and Georgiu [11], this paper applies first-digit Newcomb-Benford Law (NBL) to evaluate the reliability of the records for COVID-19 cases in all 20 Latin American countries. NBL states that the first digit is not uniformly distributed in several naturally occurring collections of numbers. Therefore, many empirical studies use the deviation from NBL as a measure of data reliability [9, 10, 12–16].

We also employ Kullback-Leibler Divergence (KLD) to compare the asymmetry among COVID-19 data reports [14]. Originally proposed by Kullback and Leibler [17], KLD is a widely used method from information theory to estimate the similarity between two probability distributions P and Q, and it is calculated by the logarithmic difference between the both probabilities. More recently, several studies have used KLD to detect anomalous observations [18, 19].

We focus on Latin America for four reasons. First, available evidence indicates that populist political leaders react more slowly to COVID-19 [15] and, according to De la Torre: “Latin America is the land of populism” [20]. Second, several socio-economic problems - such as low-quality health facilities and a high proportion of people living in slums - undermine the capacity of Latin American countries to control the spread of COVID-19 [16]. Third, skepticism about official figures can lead to ineffective policy choices [7], and political leaders in the region are especially skeptical of the destructive power of COVID-19. Finally, we find no empirical assessment of Latin American data. Most studies have applied a single methodological approach - NBL or KLD - focusing on worldwide comparisons [11, 21] or on case studies [22–24]. This study advances our current understanding on the application of statistical tools to evaluate data quality and may be easily replicated to examine health surveillance system integrity in other countries.

## Materials and methods

### Data collection

In this paper, we combine first-digit NBL and KLD to evaluate the reliability of COVID-19 records in all 20 Latin American countries using information from Our World in Data on country-level aggregate cases [25]. By reliability, we consider the “the extent to which an experiment, test or any measuring procedure yields the same results on repeated trials” [26].

### Statistical analysis

Initially proposed by Newcomb [27] and popularized by Benford [28], NBL states that some digits appear more frequently than others. Comparatively, 1 is the most common first digit, leading 30.10% of the time, and 9 is the least common, with an expected frequency of 4.58% [29]. Scholars compare observed data distribution with the theoretical expectation that the “occurrence of numbers is such that all mantissa of their logarithms are equally probable” [27]. Therefore, for the first digit,

1 $$P(d)=\left(\frac{1+d}{d}\right)\kern0.5em for\ d\in \left\{1,..,9\right\}$$

Where P(d) gives the probability of a given number occurring as the first digit. According to Hill [30], “this law implies that a number has leading significant digit 1 with probability log_10_ 2 ≅ .301, leading significant digit 2 with probability log_10_ 3 ≅ .176 and so on monotonically down to probability .046 for leading digit 9”. NBL has been used as a forensic tool to detect data irregularities in several fields, such as religious activity [31], scientific data [32], socio-economic datasets [33], electoral processes [34], international trade [35], and academic misconduct [36]. In epidemiological data, deviations from NBL may be associated with inadequate capacity in surveillance systems or intentional fraud [13].

According to Nigrini [13], in order to apply Benford’s Law to a given dataset, the data must form a geometric sequence or a number of geometric sequences for the digit pattern to conform to the NBL. In the context of COVID-19 data, the exponential growth of SARS-CoV-2 infections mets this assumption [37].

To ensure more reliable findings, we employ three goodness of fit tests (Pearson chi-square, Kolmogorov-Smirnov D statistic, and Chebyshev distance m statistic) and three conformity estimates (average mantissa, mean absolute deviation, and distortion factor). In this manner, we diminish the likelihood that our results are driven by any specific statistical technique.

The chi-square test assesses the adherence of a data set to Benford’s Law by comparing the actual and expected counts for all digits. The Kolmogorov-Smirnov (KS) test is strongly influenced by the first and second digits of the numbers and evaluates the conformity of a data set to Benford’s Law by taking into account all the digits and their actual and expected counts [13]. According to Druica, Oancea and Vâlsan [38], Chebyshev distance (MD) informs the absolute size of the difference between two distributions, and it accommodates both ordinal and quantitative variables. The Chebyshev distance is similar to the Euclidean distance and it is also known as maximum value distance [38]. Regarding conformity estimates, NBL theoretical distribution expects that the average mantissa should be .5 with variance 1/12 and skewness close to zero. The mean absolute deviation (MAD) is based on the average absolute deviation of the actual proportions from the Benford proportions [13]. MAD takes into account the expected proportions and the actual proportions for each digit, but it is not influenced by the size. According to Nigrini [13], observed values above .015 indicate nonconformity to NBL for the first digit test. Finally, the distortion factor (DF) model suggests whether data are likely to be over or underestimated [13].

We complete the analysis using KLD, a well-established measure of directed divergence in information theory [17]. Also known as relative entropy, KLD estimates how much information change it would take to encode a given distribution Q as a target distribution P. By estimating the directed divergence of two distributions, it is possible to discriminate their information and measure how similar they are. The notation for a continuous distribution is given by:

2 $$KLD\left(P\Big\Vert Q\right)=\int P(x)\mathit{\log}\frac{P_{(x)}}{Q_{(x)}} dx$$

**Fig. 1:**
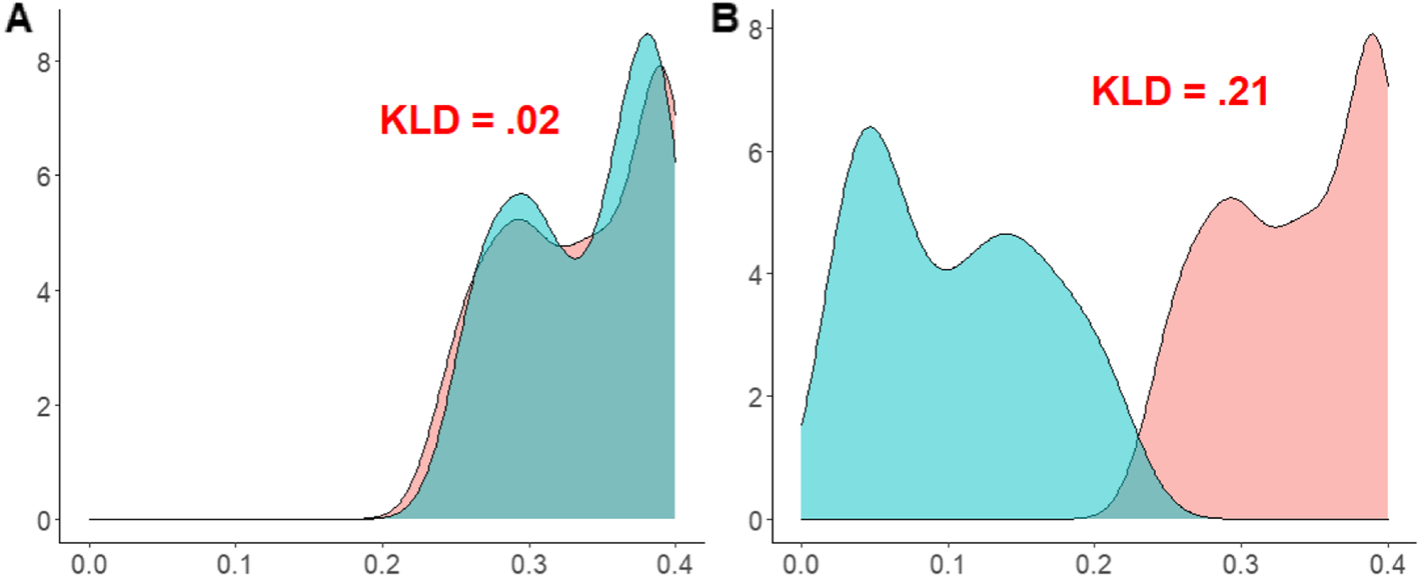
Comparing distributions with KLD in continuous distribution with different levels of entropy measured by KLD. **A** shows two probability distributions with low divergence (KLD = .02), meaning that few information changes would be required to encode p(x1) as p(x2). **B** displays two distributions with a higher divergence (KLD = .21)

Where p(x) typically represents the true distribution of data and q(x) represents a theoretical or given distribution from the same group. Originated in information theory [17], the KLD measures the expected number of extra bits required to code samples from p(x) when using a code based on q(x), rather than using a code based on p(x) [39]. KLD will always be a non-negative number without a maximum value [40]. If p(x) equals q(x), the measure will be 0, corresponding to similar distributions [41]. Figure 1 shows two pairs of distributions with different levels of entropy measured by KLD.

Figure 1A shows two probability distributions with low divergence (KLD = .02), meaning that few information changes would be required to encode p(x_1_) as p(x_2_). Figure 1B shows two distributions with a higher divergence (KLD = .21). Therefore, approximating the two data distributions would entail more information change. In addition to comparing data from the same group, KLD also applies to the estimation of pairwise divergences. KLD has been used to study outlier detection [42], sample similarity [43], SAR images [44], copying on educational tests [45], and fake news recognition [46]. Given that the number of new COVID-19 cases is a count variable, we should estimate KLD by discrete probability distribution:

3 $$KLD\left(P\Big\Vert Q\right)={\sum}_i{P}_{(i)}\mathit{\log}\frac{P_{(i)}}{Q_{(i)}}$$

Where p(x) and q(x) are two probability distributions of a discrete random variable x. Mathematically, both p(x) and q(x) sum up to 1, and p(x) > 0 and q(x) > 0 for any x in X [40]. Unlike NBL, which compares data distribution with a theoretical model, KLD does not need a priori information on distributions. It observes the direct divergences between data from similar events [14].

The reasoning to combine NBL and KLD is to strengthen the methodological rigor of our research design. While NBL is a popular tool to detect potential fraudulent activity, KLD has been used in empirical research to compare data sets, identify discrepancies between models, and measure the relative entropy between two distributions. The joint application of NBL and KLD has been used in other research areas, such as image processing [47], electrical engineering [48], and electronics [49].

### Computational tools

To estimate NBL functions, we used the *benford.analysis* package developed by Cinelli [50] and the *BenfordTests* package developed by Joenssen and Muellerleile [51], and to run KLD, we used *philentropy* package designed by Drost [52]. Statistical analyses were performed using R Statistical 4.0.4, and all significance tests were two-sided at conventional levels (*p*-value < .05).

## Results

A summary of the results from both the goodness of fit and conformity tests for new cases in Latin American countries is shown in Table 1.

**Table 1:** NBL to the number of new cases and deaths*.* Goodness of fit tests (chi_square, ks, and md statistics) and conformity estimates (mantissa, mad, and df). For the chi-square, ks and md, *p*-values below .05 indicate the that the data does not conform to Benford’s Law. Regarding conformity estimates, NBL theoretical distribution expects that the mantissa should be .5 with variance 1/12 and skewness close to zero. Mad values above .015 indicate nonconformity to NBL for the first digit test. The df model suggests whether data are likely to be over or underestimated

Variable	Country	*n*	Goodness of fit tests						Conformity estimates			
			chi_square	*p*-value chi_square	ks	*p*-value ks	md	*p*-value md	mantissa	mad	mad_conformity	df
Cases	Argentina	795	41.81	< 0.001	7.46	< 0.001	2.03	< 0.001	0.48	0.02	Nonconformity	−0.32
	Brazil	913	40.88	< 0.001	2.94	< 0.001	2.68	< 0.001	0.53	0.02	Nonconformity	3.81
	Bolivia	840	27.43	< 0.001	4.14	< 0.001	1.38	< 0.001	0.48	0.02	Nonconformity	−2.15
	Chile	959	9.59	0.29	1.1	0.06	0.89	0.08	0.51	0.01	Acceptable conformity	0.56
	Colombia	808	51.96	< 0.001	5.36	< 0.001	1.7	< 0.001	0.48	0.02	Nonconformity	−2.43
	Costa Rica	588	29.27	< 0.001	12.27	< 0.001	2.38	< 0.001	0.45	0.02	Nonconformity	−13.21
	Cuba	956	111.96	< 0.001	4.16	< 0.001	1.99	< 0.001	0.56	0.03	Nonconformity	16.32
	Dominican Republic	816	30.17	< 0.001	5.56	< 0.001	2.35	< 0.001	0.54	0.02	Nonconformity	9.32
	Ecuador	757	54.16	< 0.001	7.63	< 0.001	2.11	< 0.001	0.51	0.02	Nonconformity	7.79
	El Salvador	484	64	< 0.001	15.29	< 0.001	3.14	< 0.001	0.43	0.04	Nonconformity	−14.93
	Guatemala	946	64.1	< 0.001	4.1	< 0.001	1.38	< 0.001	0.54	0.02	Nonconformity	11.27
	Haiti	767	13.68	0.09	6.11	< 0.001	0.98	0.04	0.45	0.01	Marginally acceptable conformity	−16.97
	Honduras	622	64.27	< 0.001	11.04	< 0.001	4.01	< 0.001	0.56	0.03	Nonconformity	16.24
	Mexico	839	63.32	< 0.001	6.09	< 0.001	2.97	< 0.001	0.53	0.03	Nonconformity	5.71
	Nicaragua	139	32.1	< 0.001	26.44	< 0.001	8.47	< 0.001	0.51	0.04	Nonconformity	3
	Panama	820	16.52	0.04	4.96	< 0.001	2.16	< 0.001	0.52	0.01	Marginally acceptable conformity	5.51
	Paraguay	718	26.88	< 0.001	8.06	< 0.001	3.04	< 0.001	0.52	0.02	Nonconformity	5.24
	Peru	847	24.52	< 0.001	4.62	< 0.001	1.89	< 0.001	0.53	0.01	Marginally acceptable conformity	8.16
	Uruguay	785	21.66	0.01	5.74	< 0.001	1.4	< 0.001	0.46	0.02	Nonconformity	−11.12
	Venezuela	840	38.03	< 0.001	4.7	< 0.001	2.24	< 0.001	0.51	0.02	Nonconformity	4.59
	Latin America	978	78.95	< 0.001	4.33	< 0.001	2.18	< 0.001	0.54	0.02	Nonconformity	12.75

For all goodness of fit tests, we find significant deviations from the NBL theoretical distribution for new COVID-19 cases in most Latin American countries (*n* = 978; chi-square = 78.95; KS = 4.33, MD = 2.18; mantissa = .54; MAD = .02; DF = 12.75). Only four countries had some degree of conformity: Chile (*N* = 959; × 2 = 9.59, *p*-value = .29; KS = 1.1, p-value = .06; MD = .89, p-value = .08; mantissa = .51; MAD = .01; DF = .56), Haiti (*N* = 767; × 2 = 13.68, p-value = .09; KS = 6.11, p-value <.05; MD = .98; p-value <.05; mantissa = .45; MAD = .01; DF = − 16.97), Panama (*N* = 820; × 2 = 16.52, p-value <.05; KS = 4.96, p-value <.05; MD = 2.16, p-value <.05; mantissa = .52; MAD = .01; DF = 5.51), and Peru (*N* = 847; × 2 = 24.52, p-value <.05; KS = 4.62, p-value <.05; MD = 1.89; p-value <.05; mantissa = .53; MAD = .01; DF = 8.16).

**Fig. 2:**
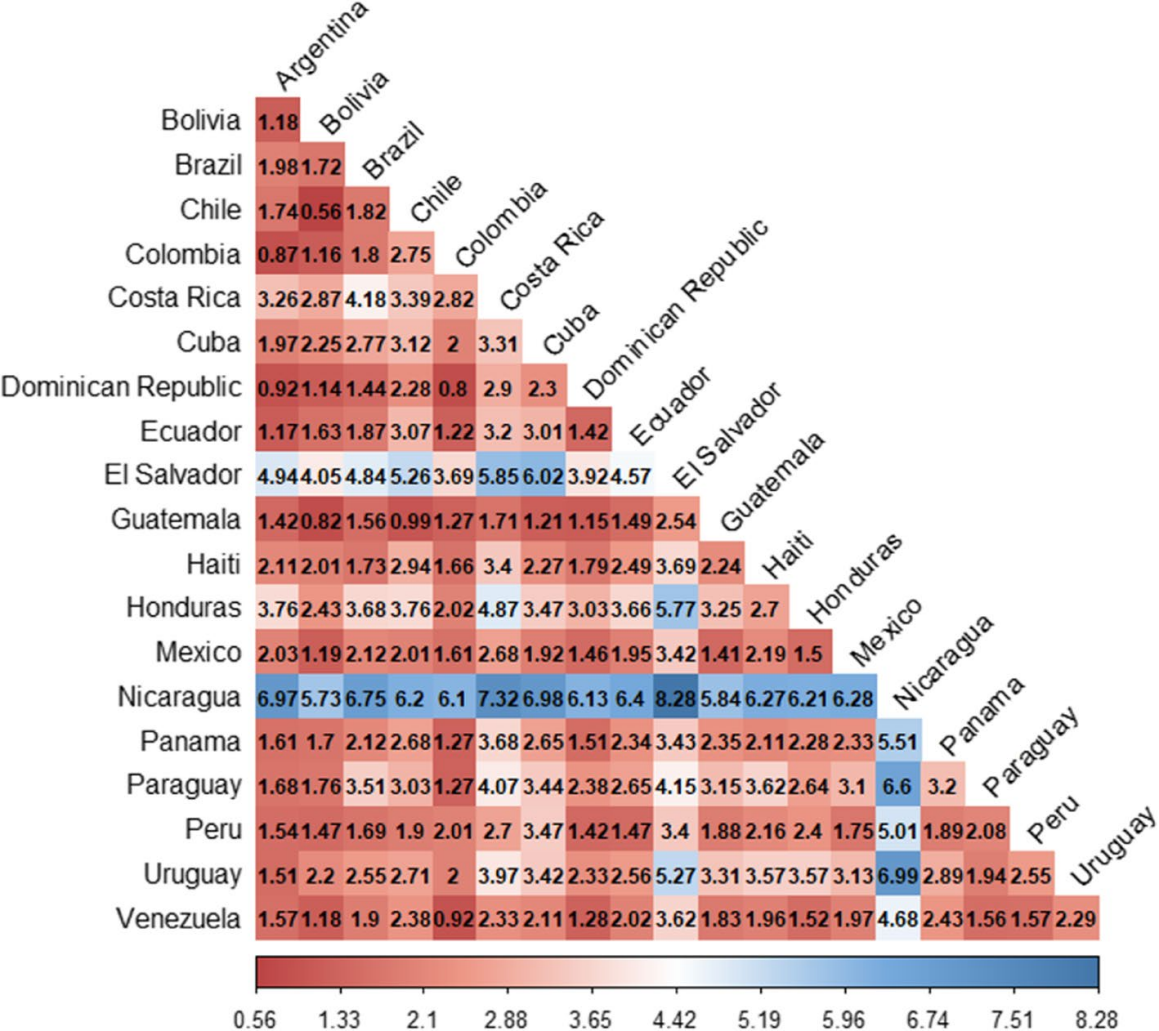
KLD pairwise comparison among Latin American countries (COVID-19 new cases). Small values indicate a low direct divergence between the two countries’ case distributions which is highlight by red color. Larger values indicate high divergence which is emphazied by the blue color

**Fig. 3:**
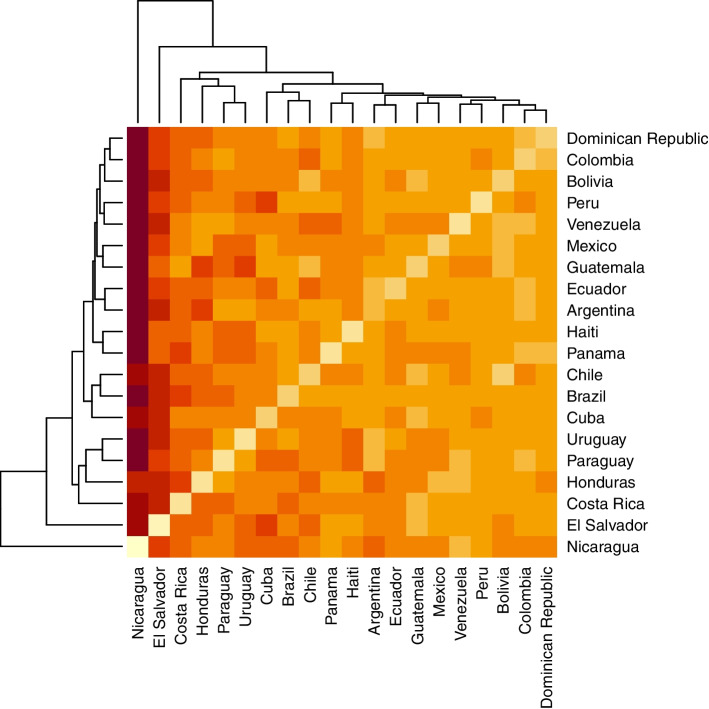
KLD heatmap across Latin American countries (COVID-19 new cases). In this plot, the more intense the red, the higher is the KLD. Analyzing the heatmap, we observe an area to the right, where the countries are more likely to present low divergences. On the other side, to the left, nations are more likely to show higher divergence. Considering the dendrogram outside the borders of the heatmap, we observe which countries are less divergent from each other

**Fig. 4:**
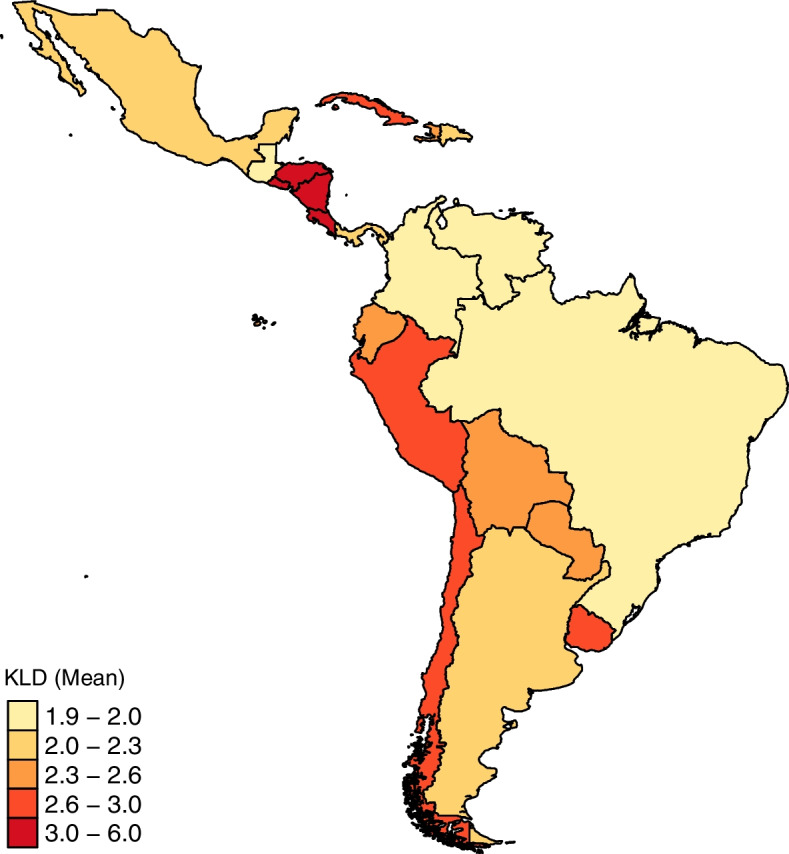
KLD map across Latin American countries (COVID-19 new cases). The darker the color, the greater the divergence level as measured by KLD. Nicaragua has the highest KLD average (6.0), which means more divergence

Figure 2 displays the KLD pairwise comparison among Latin American countries. The zero diagonal shows that a given data distribution has no direct divergence to itself. Small values indicate a low divergence between the two countries’ case distributions. Argentina to Bolivia’s KLD is 1.18, meaning that the two countries’ relative entropy is below Argentina’s median KLD which is 1.64. With few changes, it would be possible to encode data from Argentina as Bolivian records. But Argentina to Nicaragua’s KLD is 6.97, meaning that relative entropy between the two countries is significant, being the highest value in Argentina’s pairwise comparison. It would be necessary to make several changes in the data to approximate Argentina’s data to records from Nicaragua. Figures 3 and 4 depict KLD levels across Latin American countries.

In the heatmap, the more intense the red, the higher is the KLD. Analyzing the heatmap, we observe an area to the right, where the countries are more likely to present low divergences. On the other side, to the left, nations are more likely to show higher divergence. Considering the dendrogram outside the borders of the heatmap, we observe which countries are less divergent from each other. For example, Argentina is very similar to Colombia (.87), and Brazil to the Dominican Republic (1.44). Some countries only enter clusters very late after many pairs are formed, such as Nicaragua, which joins the group only after all countries have been paired. This indicates that Nicaragua’s data is very divergent to the analyzed group, even considering pairwise comparison.

The higher the divergence, the more likely the case is an outlier. The five countries with unusual distributions, that have mean KLD above the 3rd quartile value of 2.9, have also not shown conformity in NBL tests (Fig. 4). Nicaragua has the highest KLD average (6.01), which means more divergence. This can be related to differences in data collection, report or even health policies.

**Fig. 5:**
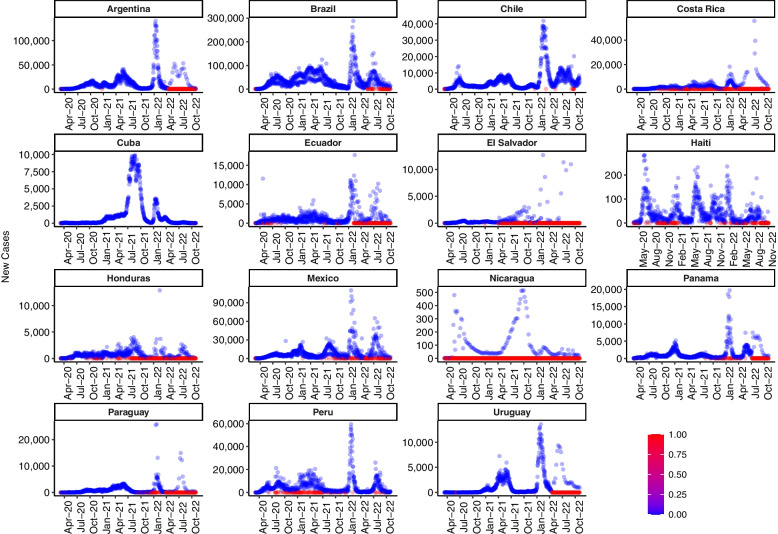
Latin American countries with higher divergence (COVID-19 new cases). The blue dots represent days with at least one new case, and the red dots represent days with zero new cases. Costa Rica, El Salvador, Honduras, and Nicaragua have a persistent occurrence of days with zero cases throughout most of the period. It is also relevant that days with many new cases are preceded and followed by days of zero cases

Once we locate the divergent countries, it is important to explore their distribution over time and try to identify patterns that can relate to the divergence. The analysis of the distributions of the countries with high mean divergence shows a pattern of recurrent days with zero new COVID-19 cases (Fig. 5).

The blue dots represent days with at least one new case, and the red dots represent days with zero new cases. Costa Rica, El Salvador, Honduras, and Nicaragua have a persistent occurrence of days with zero cases throughout most of the period. It is also relevant that days with many new cases are preceded and followed by days of zero cases. This trend is present especially in Nicaragua. To put in perspective, Nicaragua (the most divergent country), has an odds ratio of 5.87 (almost 6 days of zero new cases for every day with at least 1 case), El Salvador, the second in divergence, has an odds ratio of 1, Costa Rica (3rd) of .64 and Honduras (4th) of .55. We suspect that this pattern is due to notification delay and low testing rates.

## Discussion

Scholarly research has explored the authenticity of COVID-19 figures. Using advanced statistical tools, Kennedy and Yam [53] show that the Chinese government systematically fails to provide reliable data. More recently, Kilani and Georgiu [11] examine a sample of 171 countries and report that most of the observations exhibit suspicious patterns of data sharing.

This paper advances our understanding of the subject by applying two well-established statistical techniques to evaluate the reliability of COVID-19 records in Latin America. Under the Newcomb-Benford Law assumption, we find most countries deviate from theoretical expectations. Similarly, KLD estimates indicate that the accuracy of records is significantly heterogeneous across countries, including some abnormal observations, and one case with extreme high divergence: Nicaragua.

According to Burki [16], Nicaragua declined to close schools and shops for a significant period. More surprisingly, it was the only country in Central America to have kept open borders when the rest of the world chose to shut down the entrance of foreign people. Conversely, the COVID-19 epidemiological curve has been decreasing over time, which makes us doubt the integrity of the health surveillance system in Nicaragua. With only 18,400 confirmed cases and 225 deaths registered by November 4, 2022, Nicaragua is an extreme case of unreliable data. These findings are supported by recent scholarly publication that data from autocratic regimes are less reliable and should be treated with more caution [10, 54].

Notification delay has been a concern in Latin America from the beginning [55], and is documented in different studies [56]. According to Our World in Data, there is a strong positive correlation between the daily report of new cases and day-to-day test execution [25]. Other studies also find an association between daily tests performed and daily notifications of new cases. The lack of testing affects COVID-19 tracing [57], monitoring [58], and evaluation [59].

Latin American countries faced severe problems in managing the COVID-19 crisis. In addition to the lack of transparency in handling and sharing data, many political leaders downplayed the destructive power of SARS-CoV-2. For instance, Brazilian president Jair Bolsonaro repeatedly denied social distancing as a preventive measure [60]. In Mexico, one of the most affected countries worldwide with more than 320,000 deaths on November 4, 2022, president Andrés Manuel López Obrador called COVID-19 “not even as bad as the flu” [16].

On the one hand, these results enhance our knowledge of statistical tools and may be easily replicated to examine epidemiological data in other countries, being able to monitor aspects such as notification delay. On the other, we need to investigate how countries with such different social and economical characteristics (Chile and Haiti, for example) manage to obtain the same degree of data conformity. Search for which factors can produce this phenomenon is a challenge for future research agenda.

Our findings have significant implications for global and public health policy and practice. The results of the study provide important insights into the role of reliable data on evidence based public policy. The study also provides guidance for practitioners, policy makers, and other stakeholders regarding the best practices for detecting data inconsistencies. Overall, the findings of the current study can help to inform and shape future public health efforts, and can ultimately lead to better health outcomes.

Finally, the scientific examination of COVID-19 data is hampered by a number of weaknesses. First, data may not be collected accurately or consistently, leading to incorrect or incomplete results. Additionally, there is a lack of standardization across countries, which can lead to discrepancies between results. Finally, the data analysis may be subject to bias, either from the researcher or from external factors. We tried to ameliorate this shortcoming by providing full access to datasets and computational scripts.

## Conclusions

Valid and reliable data is key to effective public policy. If information is flawed, government intervention no longer accomplishes its desired purposes. In this paper, we provide evidence that COVID-19 records in Latin America are likely to deviate from NBL, which is a widely employed tool to spot data inconsistencies. In addition, we find high levels of heterogeneity among countries regarding figures reliability, according to KLD estimates. Nicaragua, for instance, is an example of an extreme case of unreliable data. A limitation of our study is the focus on only one specific geographical region. Future scholarly research can investigate the extent to which epidemiological data in other periods and for different countries conform to the unified framework we developed by combining NBL and KLD in the same reproducible research design.

## Data Availability

Replication materials, including raw data and computational scripts, are available on <https://osf.io/efw93/>.

## Abbreviations

COVID-19: disease caused by the SARS-CoV-2
DF: distortion factor
KLD: Kullback-Leibler Divergence
KS: Kolmogorov-Smirnov
MAD: mean absolute deviation
MD: Chebyshev distance
NBL: Newcomb-Benford Law
SAR: aperture radar
